# Detection and Phylogenetic Characterization of Human Hepatitis E Virus Strains, Czech Republic

**DOI:** 10.3201/eid1705.101205

**Published:** 2011-05

**Authors:** Petra Vasickova, Michal Slany, Pavel Chalupa, Michal Holub, Radek Svoboda, Ivo Pavlik

**Affiliations:** Author affiliations: Veterinary Research Institute, Brno, Czech Republic (P. Vasickova, M. Slany, I. Pavlik);; Charles University in Prague and University Hospital Bulovka, Prague, Czech Republic (P. Chalupa, M. Holub);; Faculty of Medicine Masaryk University and the Faculty Hospital Brno, Brno (R. Svoboda)

**Keywords:** acute hepatitis E, viruses, hepatitis, phylogenetic analysis, zoonosis, public health, dispatch

## Abstract

To determine the origin of hepatitis E virus in the Czech Republic, we analyzed patient clinical samples. Five isolates of genotypes 3e, 3f, and 3g were obtained. Their genetic relatedness with Czech strains from domestic pigs and wild boars and patient recollections suggest an autochthonous source likely linked to consumption of contaminated pork.

Hepatitis E virus (HEV) is a leading cause of epidemics and sporadic cases of enterically transmitted hepatitis worldwide. The zoonotic potential of HEV was recognized recently, and pigs and other animal species were considered natural reservoirs for the virus (*1*). Currently, mammalian HEV strains segregate into 4 major genotypes. The relative conservation of genotypes 1 and 2 corresponds to their primary circulation within humans. Genotype 1 consists of epidemic strains from developing countries in Asia and Africa, and representatives of genotype 2 have been described in Mexico and African countries. The diversity of genotypes 3 and 4 is related to their origin from a variety of animal species. Genotype 3 is widely distributed and has been isolated from patients with sporadic cases of acute hepatitis E worldwide. Genotype 4 contains strains of human and animal origin, especially in isolates from Asian countries (*2**,**3*).

In the Czech Republic, hepatitis E incidence has been increasing since the first case was described in 1996. From 1996 through 2005, a total of 159 cases of HEV infection were reported; 23% of those cases were associated with travel to industrialized countries (*4*). In 2005, 37 hepatitis E patients were reported in the Czech Republic, while in 2009 the number increased to 99 (*5*). On the basis of these data, extensive genomic variability among HEV isolates and their known geographic distribution, we conducted a phylogenetic analysis of HEV isolates from clinical samples of Czech patients with acute hepatitis E to determine the origin of the infection.

## The Study

Stool samples from a total of 10 patients with serologically confirmed acute hepatitis E were tested (online Appendix Table, www.cdc.gov/EID/content/17/5/917-appT.htm). Informed consent was obtained from all patients involved in this study (Ethics Committee, University Hospital Bulovka; IRB00002721).

Two hundred and fifty milligrams of stool sample was suspended in 2.25 mL of phosphate-buffered saline, homogenized by vortexing, and clarified by centrifugation at 3,000× *g* for 15 min. RNA was extracted from 140 μL of supernatant by using the QIAamp Viral RNA Kit (QIAGEN, Hilden, Germany), according to the manufacturer’s instructions. A positive template control was isolated from swine feces positive for HEV RNA from a previous study (*6*), and a water sample was included as a negative control.

Detection of HEV RNA was performed by nested reverse transcription-PCR with 2 sets of degenerate primers that targeted open reading frame 1 (ORF1) and the overlapping part of ORF2 and ORF3 (ORF2/3) of the HEV genome as described previously (*6*). Nondiluted and 10-fold diluted samples of isolated RNA were analyzed. Subsequently, the specific PCR product (length 242 bp, ORF1 primers) obtained from 2 independent RNA isolations was prepared for sequencing by the QIAquick PCR Purification Kit (QIAGEN). Both strands were sequenced at Eurofins MWG Operon (Ebersberg, Germany).

The sequencing and phylogenetic analysis were carried out by using MEGA version 3.1 software (www.megasoftware.net). The method of neighbor-joining with 1,000 replications in the bootstrap test was used for phylogenetic analysis (*7*), and bootstrap values >50% were considered significant. The 5 most similar HEV sequences available in GenBank database were chosen for each presented Czech human HEV isolate (isolate CZhHEV), according to the BLAST algorithm (http://blast.ncbi.nlm.nih.gov/Blast.cgi). Selected sequences were supplemented with representatives of genotype 3e (strain G2, GenBank accession no. AF110389), 3f (strain G1, accession no. AF110388), and 3g (strain Osh 205, accession no. AF455784) (*2*).

HEV RNA was detected in clinical samples from 6 of 10 patients whose samples were tested. Results obtained by using primers specific for ORF1 and ORF2/3 of the HEV genome were in agreement, except for material from 1 patient. Specific PCR products of 5 CZhHEV isolates were sequenced (Table A1). The sequence analysis showed that sequences of CZhHEV isolates shared a homology of 81.4% (CZhHEV107-09 and CZhHEV113-09) to 100.0% (CZhHEV113-09 and CZhHEV114-09).

Phylogenetic analysis showed that isolates CZhHEV107-09 and CZhHEV197-09 were most genetically similar to subtype 3e (strain G2) and were clustered together with sequences from a pig from Hungary (HUN-072, accession no. EU718650), a person from Germany (V0713286, accession no. EU7879117), pigs from Japan (swJ8–5, accession no. AB248521; swJ12–4, accession no. AB248522; swJB-E10, accession no. AB481226), and a patient from Japan (JNH-Ehi04L, accession no. AB291958). Isolate CZhHEV107-09 had highest sequence homology (88.8%) with strain HUN-072, while isolate CZhHEV197-09 and Greek strain G2 shared homology of 93.0%.

Isolate CZhHEV108-09 belonged to the subtype 3g, together with other Czech strains from wild boars (CZwb51-09, accession no. GU299814; CZwbHEV71-09, accession no. GU299816), Czech domestic pigs (CZswHEV21, accession no. EU117413; CZswHEV6, accession no. EU117410), German strain V0714229 (GenBank accession no. EU879118) and strain Osh205. CZhHEV108-09 shared highest homology (91.3%) with strain CZwbHEV51-09 and strain V0714229.

Identical sequences of CZhHEV113-09 and CZhHEV114-09 isolates were clustered to subtype 3f (strain G1). A human strain from Spain (VH2, accession no. AF195065), swine strains from the Netherlands (NLSW28, accession no. AF336003; NLSW82, accession no. AF336009) and from Spain (SWP6; accession no. EU723514) also belonged in subtype 3f. The highest sequence homology (95.0%) was found for isolates CZhHEV113-09, CZhHEV114-09, and strain VH2 (Figure). Sequences of isolates CZhHEV107-09, CZhHEV108–09, CZhHEV113-09, and CZhHEV197-09 have been deposited in GenBank under accession nos. GU299817, GU299812, GU299813, and GU299815, respectively.

**Figure:**
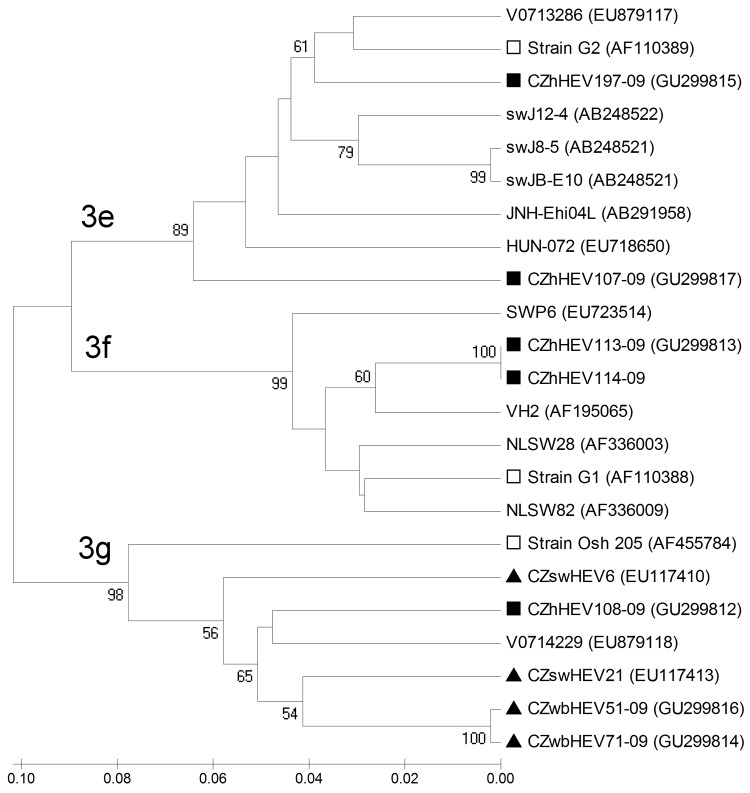
Phylogenetic tree constructed with MEGA version 3.1 software (www.megasoftware.net) by using the neighbor-joining method with 1,000 replication in bootstrap test based on 242-bp–long sequences within open reading frame 1 (OFR1) of hepatitis E virus (HEV) isolates and only bootstrap values (percentages) >50 are indicated on the tree. Key: ■, sequences originating from 5 Czech patients; □, representatives of genotype 3 subtypes: 3e (strain G2), 3f (strain G1), and 3g (strain Osh 205); and the 5 HEV strains most similar to human isolates from the Czech Republic: human strains from Germany, V0713286 and V0714229; swine strains from Japan, swJ12–4, swJ8–5, and swJB-E10; human strain from Japan, JNH-Ehi04L; swine strain from Hungary, HUN-072; swine strains from the Netherlands, NLSW28 and NLSW82; swine strain from Spain, SWP6; human strain from Spain, VH2; ▲, strains from the Czech Republic originating from domestic pigs, CZswHEV6 and CZswHEV21; and from wild boars, CZwbHEV51–09 and CZwbHEV71–09. GenBank accession numbers of chosen sequences are included in the phylogenetic tree. Scale bar indicates nucleotide substitutions per site.

## Conclusions

We tested stool samples of 10 patients with serologically confirmed acute hepatitis E and, despite this confirmation, have detected HEV RNA in only 6 of these patients (Table A1; Figure). Most serologic assays for diagnosing hepatitis E use recombinant proteins of genotypes 1 and 2, and these tests may be less sensitive and specific for detection of genotypes 3 and 4 of genus *Hepevirus* (*8**,**9*). These results were confirmed by a study performed in the Czech Republic, which showed that 28 (47.5%) of 59 IgM anti-HEV positive cases were in fact false positives (unpub. data).

According to Lu et al. (*2*), subtypes 3c, 3e, 3f, 3h, and 3i have been mainly identified in Europe and subtype 3g in Asia. In our study, isolates CZhHEV107-09 and CZhHEV197-09 were genetically related to subtype 3e, while identical CZhHEV113-09 and CZhHEV114-09 belonged to subtype 3f. Isolate CZhHEV108-09 clustered with subtype 3g and a strain from a wild boar from the Czech Republic (CZwbHEV51-09) and a strain from a human in Germany that was associated with consumption of wild boar meat, offal, and locally produced meat products (*10*). These findings supported the idea of zoonotic transmission of HEV. Moreover, other Czech strains from domestic pigs belonged also in subtypes 3g and 3f (Figure).

The patient from whose stool isolate CZhHEV108-09 was identified reported eating homemade pig-slaughter products 1 month before the first symptoms of hepatitis E and also visiting sushi bars in Germany that served grilled pork. As Lupulovic et al. (*11*) reported, the prevalence of anti-HEV immunoglobulin (Ig) G in pigs raised in family backyards is similar to the prevalence in those bred on commercial farms. Thus, infection obtained during home slaughter is probable. On the other hand, Germany is one of the biggest exporters of domestic pigs and pork meat into the Czech Republic. Based on geographic closeness, circulation of subtype 3g between the 2 countries is possible. Moreover, that HEV infection resulted from the consumption of insufficiently heat-treated meat in sushi bars cannot be excluded.

The identity of sequences of isolates CZhHEV113-09 and CZhHEV114-09 has strongly hinted at an identical source of HEV. Both patients lived in the same city and had overlapping times of hospitalization. Furthermore, the patients reported consumption of pork and that they used the same knife and chopping board for raw and cooked meat (Table A1). Therefore, cross-contamination during the meat processing is probable.

## Appendix

**Table A1:** Characterization of the 10 patients tested in the study and detection of HEV RNA by nested RT-PCR*

Patient no.	Age, y/sex	Town of origin	Possible risk factors	Bilirubin, μmol/L	ALT, μkat/L	AST, μkat/L	GGT μkat/L	ALP, μkat/L	Systemic disease	Hospital care, d	Isolate name	ORF1†	ORF2/3
1‡	62/M	A	NS	152.00	38.40	19.30	5.60	4.83	NS	13	CZhHEV107-09		
2‡	66/M	B	Ate homemade slaughter products; used same knife and chopping board for raw and cooked meat; ate undercooked meat in sushi bar	110.00	40.78	8.72	15.37	4.73	NS	11	CZhHEV108-09	+	+
3‡	62/M	B	Used same knife and chopping board for raw and cooked meat	614.00	25.22	28.78	4.98	5.77	DM, IHD	41	CZhHEV113-09	+	+
4‡	75/M	B	NS	203.00	20.24	28.57	2.68	3.35	NS	15	CZhHEV117-09	+	+
5‡	77/F	B	Ate pork	77.00	78.87	51.78	8.92	6.92	NS	10	CZhHEV114-09	–	+
6‡	59/M	B	NS	297.00	88.44	45.57	4.52	2.50	CLL	10	ND	+	+
7‡	59/M	C	Ate raw meat	14.50	8.99	5.52	1.96	2.24	NS	8	ND	–	–
8§	65/F	C	NS	36.30	2.32	0.90	8.76	4.83	BC, CH	11	ND	–	–
9§	27/F	C	Visited China	5.80	1.89	2.08	1.03	1.40	NS	7	ND	–	–
10§	53/M	C	NS	113.10	19.48	20.43	20.52	10.49	SM	14	CZhHEV197-09	–	–

*HEV, hepatitis E virus; RT-PCR, reverse transcription–PCR; ALT, alanine aminotransferase; AST, aspartate aminotransferase; GGT, gama-glutamyltransferase; ALP, alkaline phosphatase; ORF1, primers targeting open reading frame 1 of HEV genome; ORF2/3, primers targeting overlapping part of HEV ORF2 and ORF3; NS, not shown; DM, diabetes mellitus; IHD, ischemic heart disease; CLL, chronic lymphatic leukemia; ND, not detected; BC, breast cancer; CH, chronic hepatopathy; SM, smoldering myeloma. †Specific PCR product sequenced. ‡Detection of immunoglobulin (Ig) G antibodies against HEV by EIAgen HEV IgM kit (Adaltis, Rome, Italy), EIAgen HEV IgG Kit (Adaltis) and Recomblot HEV IgM/IgG (Microgen GmbH, Neured, Germany). §Detection of IgM antibodies against HEV by HEV IgM ELISA 3.0 (MP Diagnostics, Illkirch Cedex, France) and HEV ELISA (MP Diagnostics).
